# Supplementary material for: A Self-Powered High-Responsivity, Fast-Response-Speed Solar-Blind Ultraviolet Photodetector Based on CuO/β-Ga2O3 Heterojunction with Built-In Potential Control
Source: Nanomaterials (Basel). 2023 Mar 6;13(5):954. doi: 10.3390/nano13050954 (PMC10005126; doi:10.3390/nano13050954)
Supplement: Supplementary file 1 [file nanomaterials-13-00954-s001.zip › nanomaterials-2265963-supplementary.pdf]

Article

A Self-Powered High-Responsivity, Fast-Response-Speed Solar-Blind Ultraviolet Photodetector Based on CuO/β-Ga_2O_3 Heterojunction with Built-In Potential Control

Sangbin Park, Younghwa Yoon, Hyungmin Kim, Taejun Park, Kyunghwan Kim and Jeongsoo Hong *

Department of Electrical Engineering, College of IT Convergence, Gachon University, 1342, Seongnam-daero, Sujeong-gu, Seongnam-si 13120, Gyeonggi-do, Republic of Korea; tkdrns7@naver.com (S.P.); yh7290@naver.com (Y.Y.); kjf9252@gmail.com (H.K.); mkn12123@naver.com (T.P.);
khkim@gachon.ac.kr (K.K.)
* Correspondence: hongjs@gachon.ac.kr

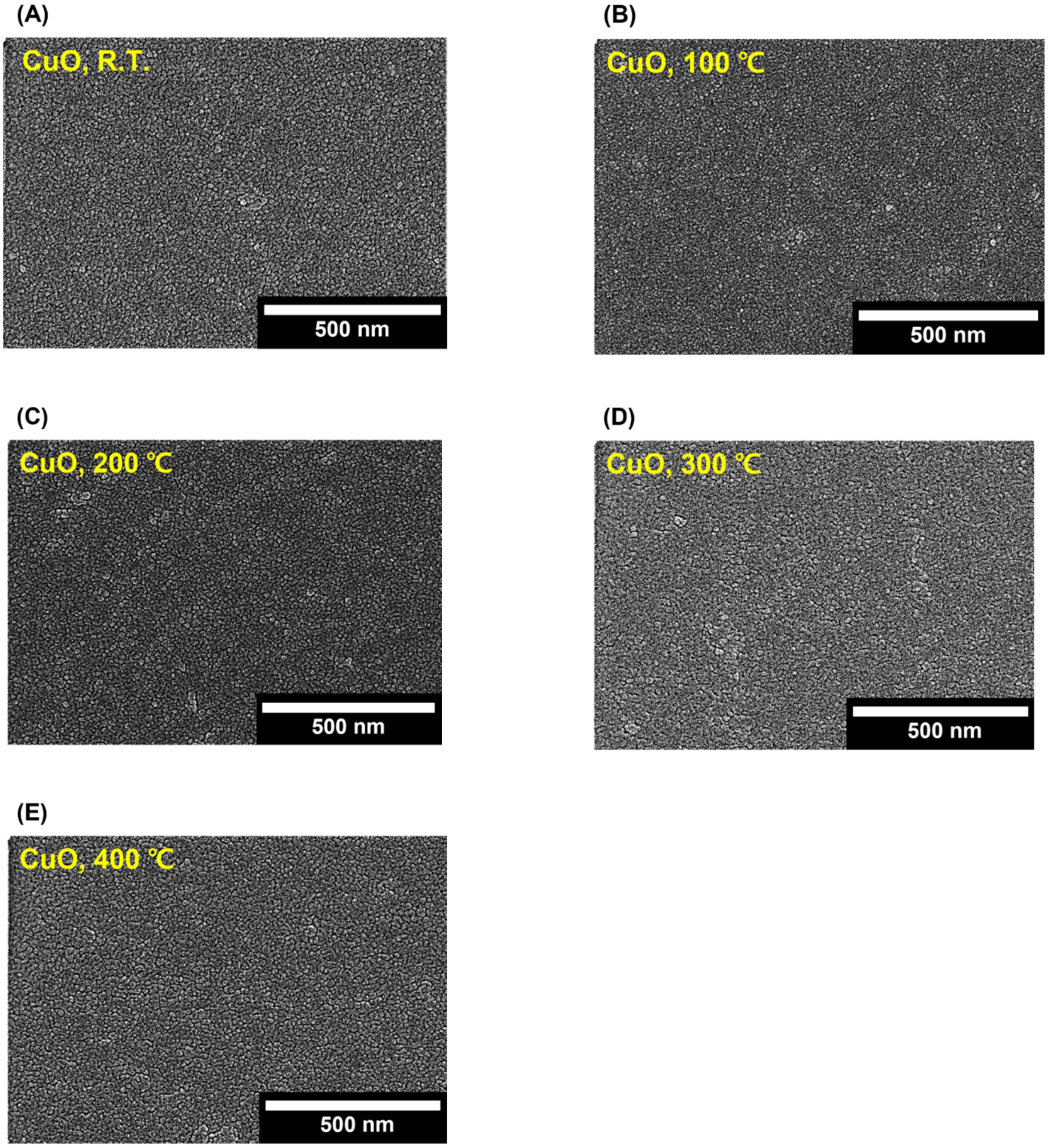

Figure S1. SEM images of surface morphology of CuO film deposited on the glass substrate, The plan-view images of a) as-fabricated CuO film at room temperature and post-annealed at b) 100 °C; c) 200 °C; d) 300 °C; e) 400 °C.

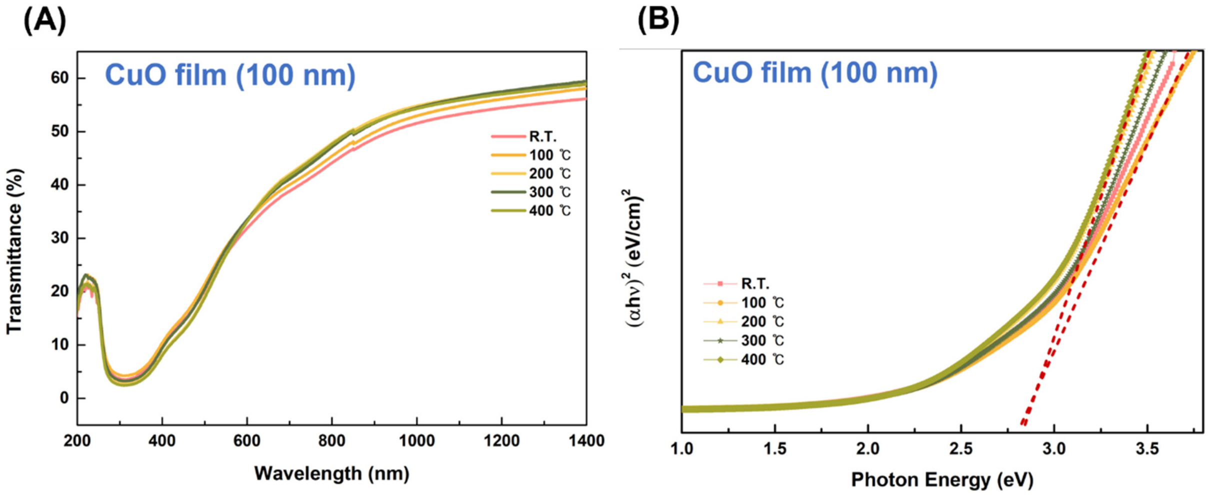

Figure S2. The optical properties of the as-fabricated CuO film at room temperature and different post-annealing temperatures, which deposited on the glass substrate. (a) The transmittance and (b) optical band gap energy of as-fabricated CuO films at room temperature and different post-annealing temperatures.

Table S1. The optical and electrical properties for the CuO film and β-Ga_2O_3 wafer.

Parameter	Properties	
Layer	CuO	β-Ga_2O_3
Optical bandgap energy (eV)	2.80	4.80
Work function (eV)	6.55	5.65
Electron affinity (eV)	5.02	4.00

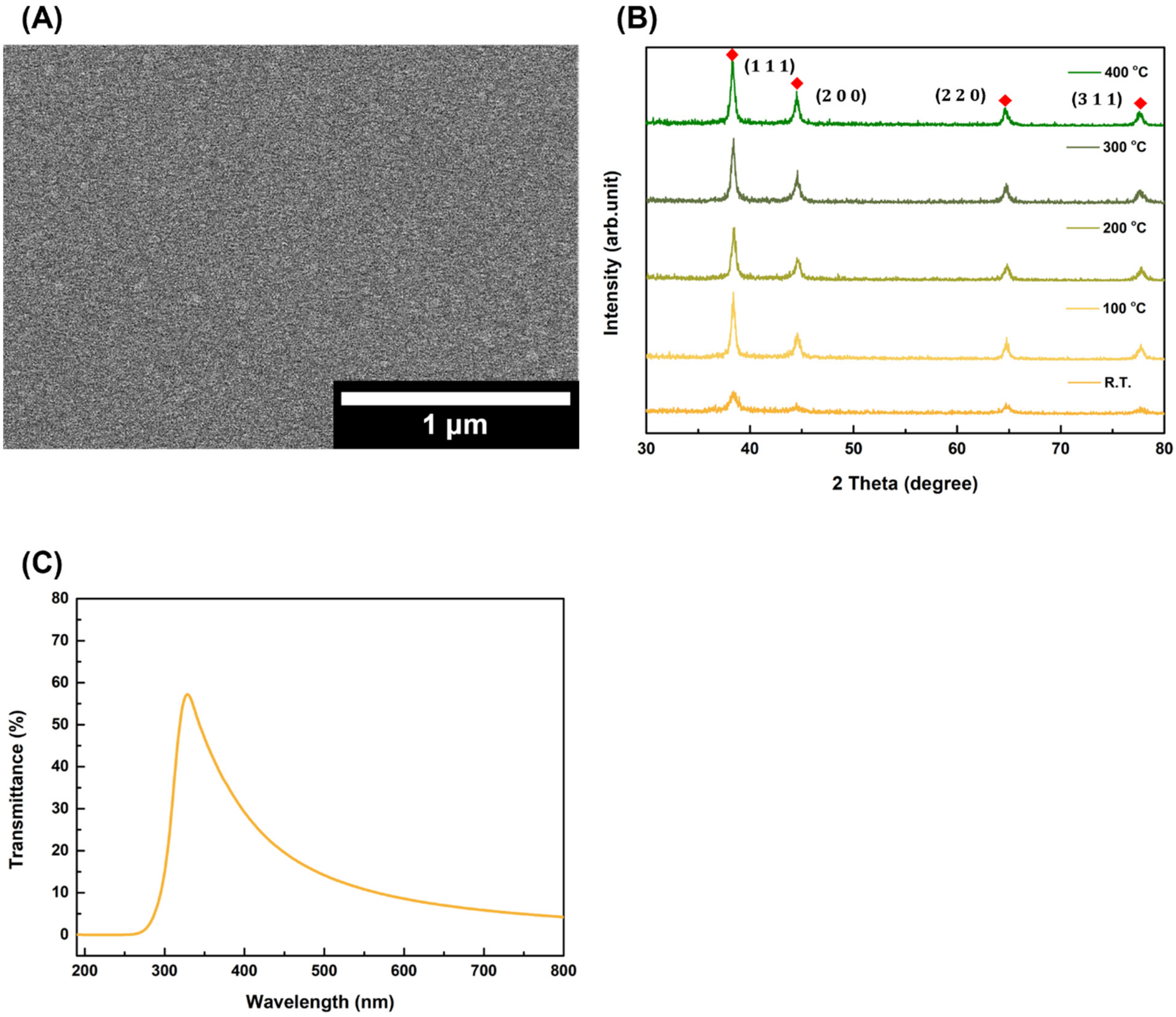

Figure S3. The structural and optical properties of 20-nm Ag electrode deposited on the glass substrate. (a) SEM plan-view images of 20-nm Ag electrode. (b) XRD pattern of 20-nm Ag thin film at room temperature and different post-annealing temperatures. (c) The transmittance of Ag electrode deposited on the glass substrate.

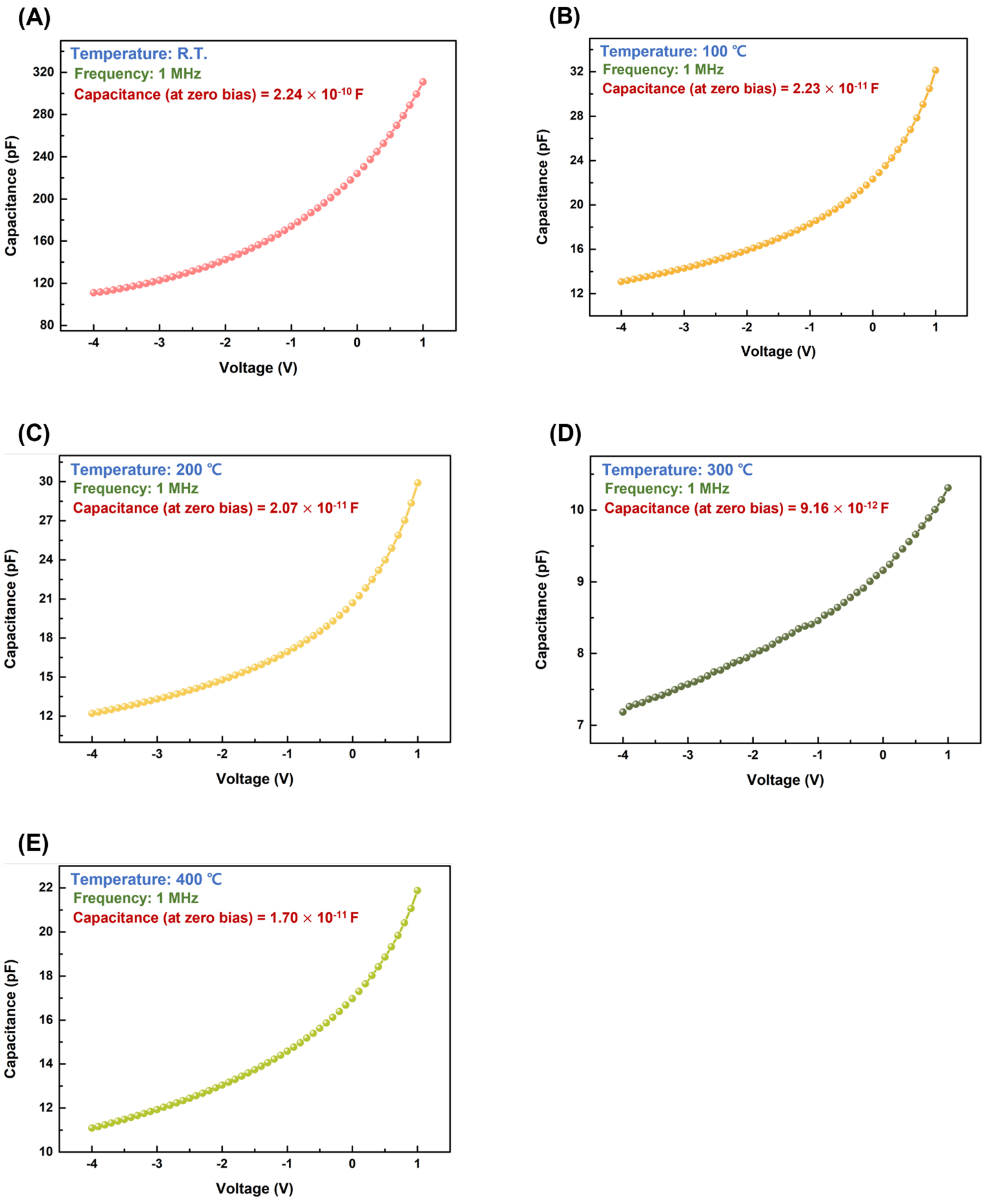

Figure S4. Capacitance-voltage (C-V) characteristic of as-fabricated photodetector at (a) room temperature and devices post-annealed at (b) 100 ℃; (c) 200 ℃; (d) 300 ℃; (e) 400 ℃ ranging from -4 V to 1 V.

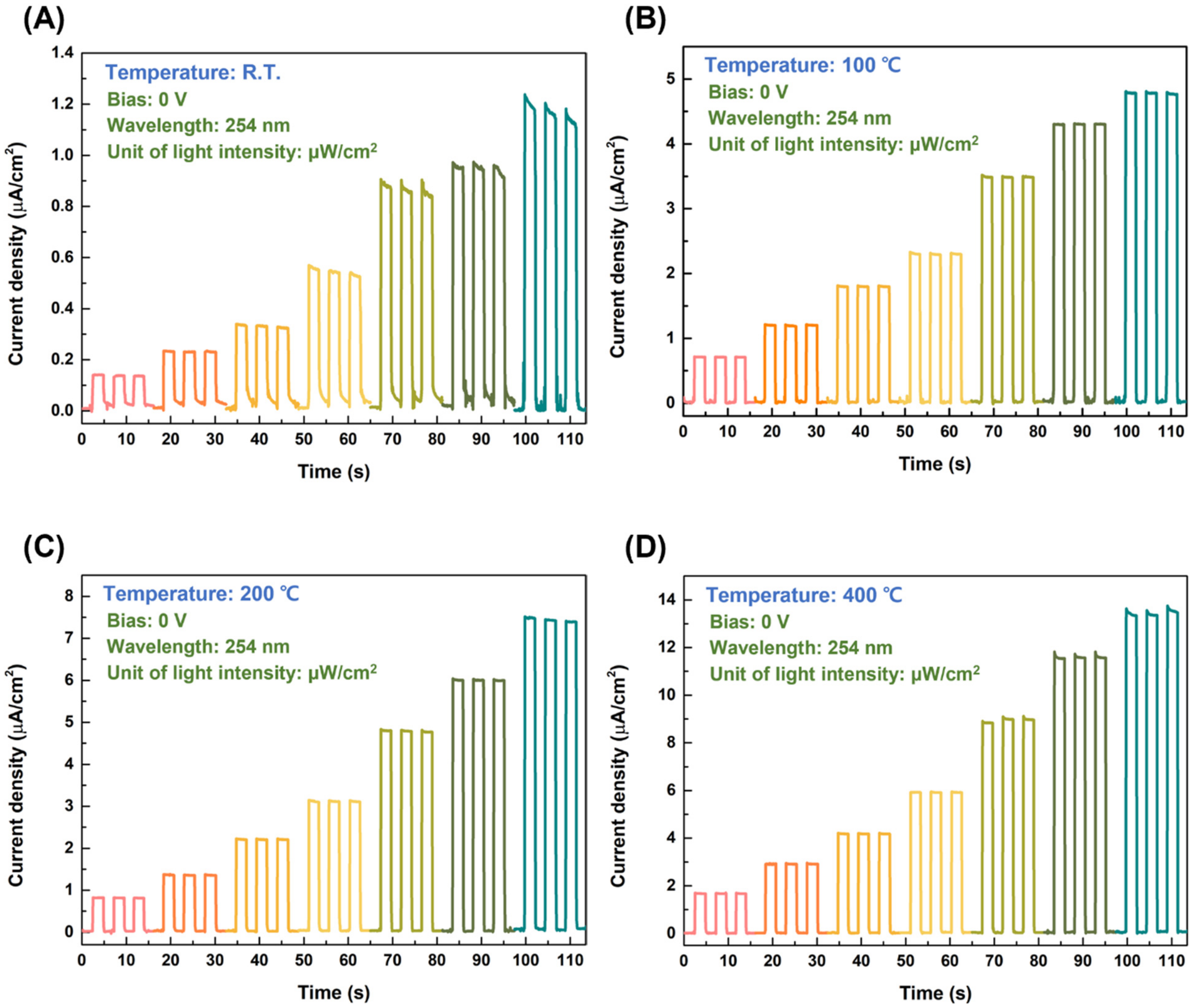

Figure S5. Time-dependent photocurrent density characteristic of as-fabricated photodetector at (a) room temperature and post-annealed at (b) 100 ℃; (c) 200 ℃; (d) 400 ℃ under 254-nm solar-blind UV light with various light intensities.

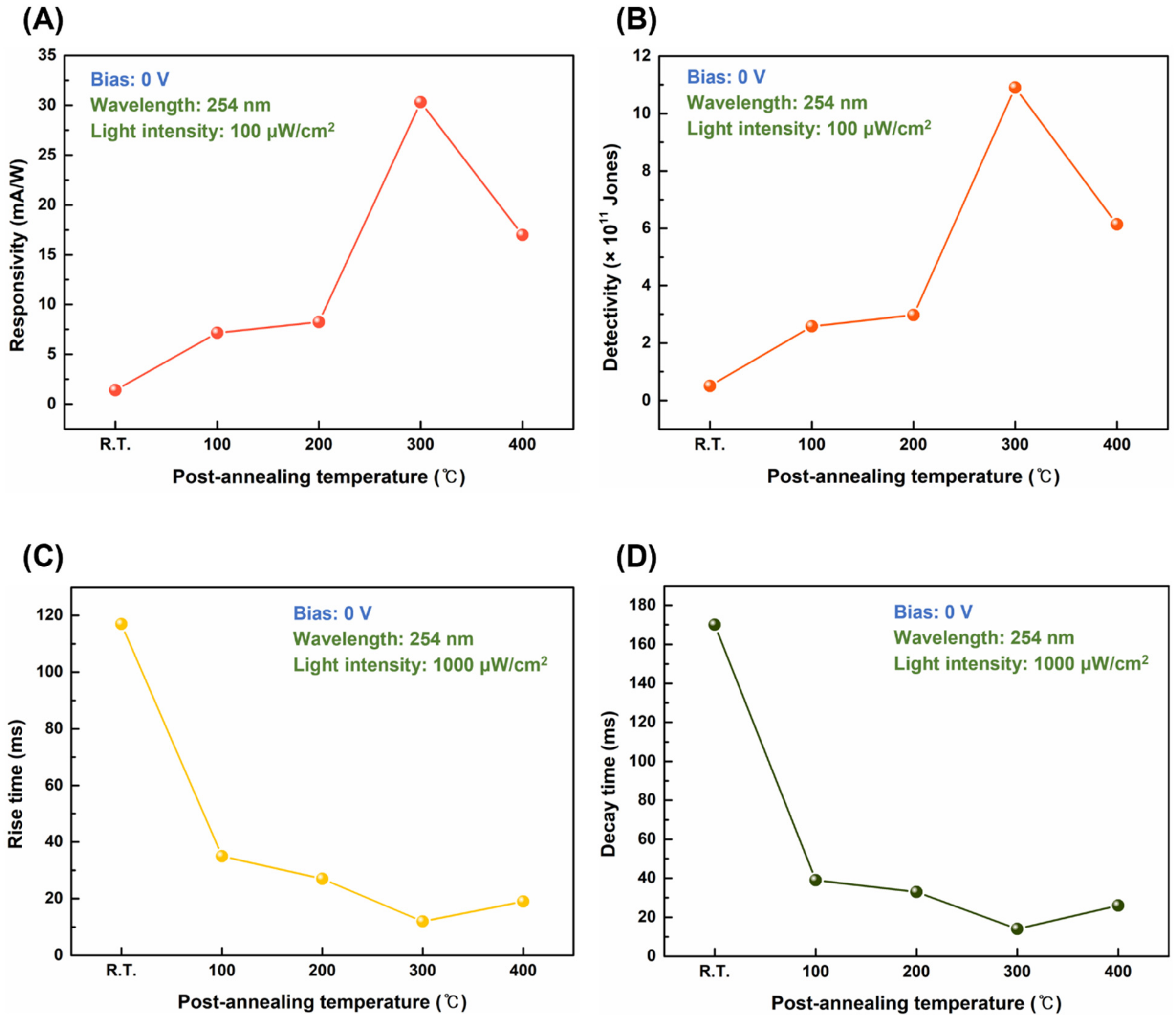

Figure S6. The post-annealing temperature-dependent (a) responsivity and (b) detectivity of the photodetector under 254-nm UV light with a 100 μW/cm^2 light intensity; (c) Rise time and (d) decay time of the photodetector with different post-annealing temperatures under 254-nm UV light with a 1000 μW/cm^2 light intensity

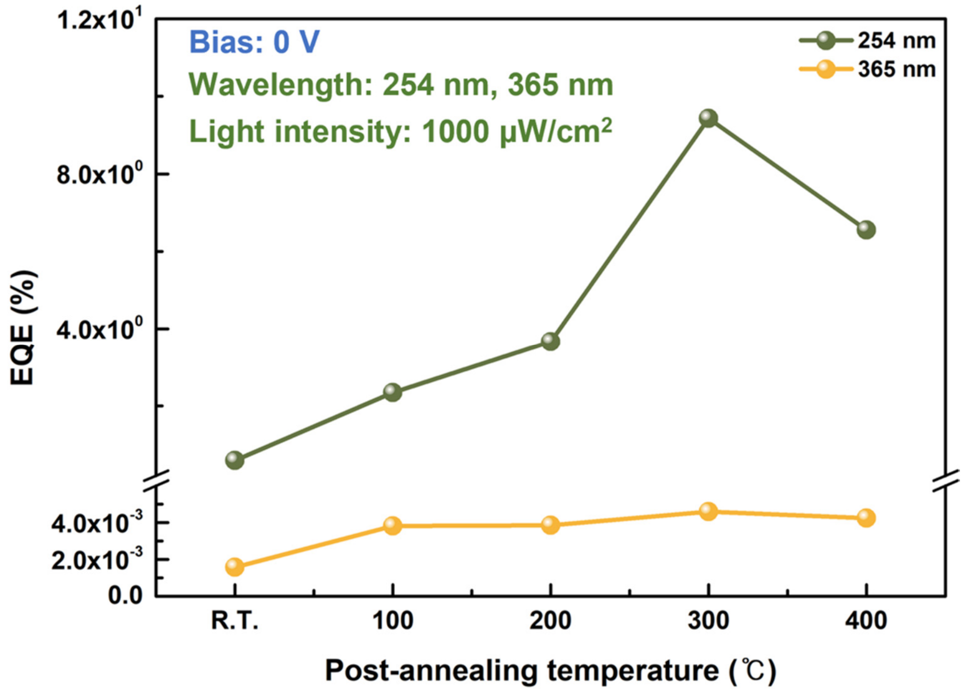

Figure S7. The post-annealing temperature-dependent EQE under the 254-nm solar-blind UV and 365-nm UV-A light with a 1000 μW/cm^2 light intensity.

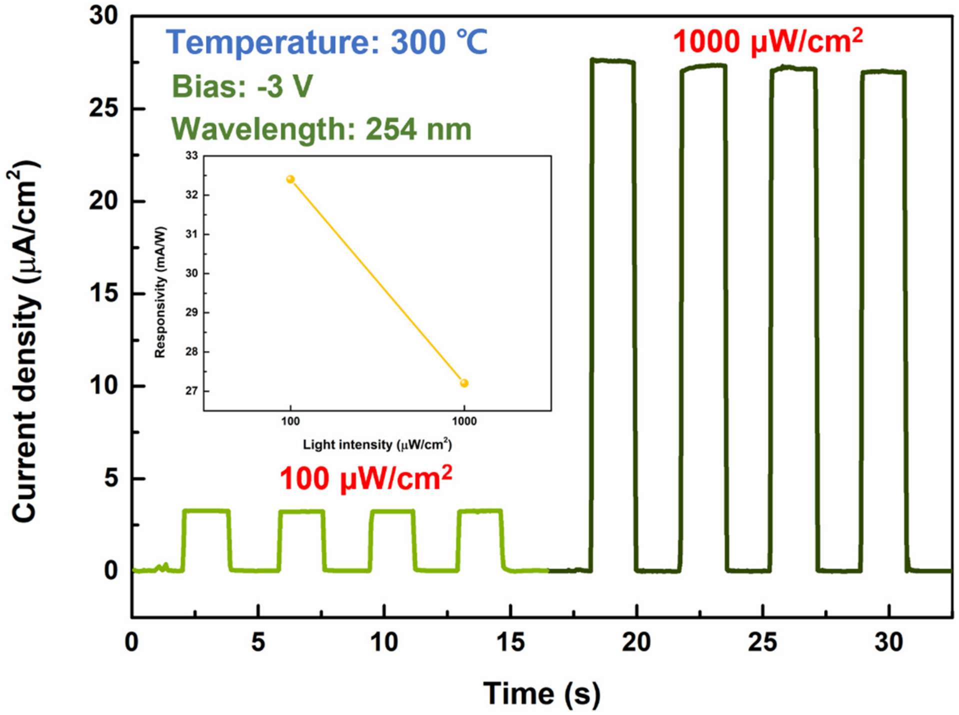

Figure S8. Time-dependent photocurrent density characteristics and responsivity of photodetector post-annealed at 300 °C under 254-nm solar-blind UV light with light intensity of 100 μW/cm^2 and 1000 μW/cm^2.
